# Comparing automated and manual assessments of tear break-up time using different non-invasive devices and a fluorescein procedure

**DOI:** 10.1038/s41598-024-52686-0

**Published:** 2024-01-30

**Authors:** Fabrizio Zeri, Giulia Carlotta Rizzo, Erika Ponzini, Silvia Tavazzi

**Affiliations:** 1 Department of Materials Science, University of Milano-Bicocca, Via Roberto Cozzi, 55, 20125 Milan, Italy; 2 COMiB Research Centre in Optics and Optometry, University of Milano-Bicocca, Milan, Italy; 3 College of Health and Life Sciences, Aston University, Birmingham, UK

**Keywords:** Lacrimal apparatus diseases, Eye manifestations

## Abstract

To evaluate the agreement and repeatability of an automated topography-based method for non-invasive break-up time (NIBUT) analyses in comparison with two other NIBUT procedures, the fluorescein procedure (fBUT), and with the manual assessment with the same device. In the first experiment, a semi-randomised crossover study was performed on forty-three participants (23.1 ± 2.1 years). NIBUT measurements were collected in a randomised order, in both eyes of participants with EasyTear View + (Easytear, Rovereto), Polaris, and Sirius + (CSO, Firenze). Then a fBUT was collected. The overall measurement procedure was repeated in a further session (retest) on the same day. In a second experiment, a retrospective randomised crossover study was performed on eighty-five NIBUT videos previously recorded by the Sirius+. Two observers assessed manually the videos and the NIBUTs were compared with the automatic ones. In the first experiment, ANOVA showed a significant difference between the four measures in both eyes (*p* < 0.001). Significant differences were found in the paired comparisons between each NIBUT procedure and fBUT (Wicoxon; *p* < 0.05). Sirius+ resulted in agreement only with Polaris in the left eye. Correlations between all NIBUT procedures resulted in statistical significance in both eyes. All procedures showed very good test-rest reliability. In the second experiment, a significant correlation between automated and manual NIBUT was found, but also a significant statistical difference between the two measurements, although clinically negligible (0.3 s). The investigated NIBUT devices perform differently from each other (and from fBUT), so they cannot be considered interchangeable. The automated measure of NIBUT with Sirius+ has a negligible clinical difference compared to manual assessment on the same device.

## Introduction

The tear film is a thin structure (about 2.0–5.5 µm thick^1,2^), extremely sophisticated in functioning and composition with a crucial role in maintaining ocular surface physiology. The assessment of tear film is of paramount importance in diagnosing dry eye disease (DED)^3^, One aspect of the tear film which is crucial to investigate for DED diagnosis is its stability^3–5^. Many factors determine the stability of tear film such as a sufficient and balanced production of the main components, which have to be spread efficiently on the ocular surface by the blinking system^5^. According to the three-layered model of the tear film, the stability is maintained by the prevention of evaporation by the outer lipid layer, the increase of volume and lubricity by the aqueous layer, and the reduction of hydrophobicity of the corneal epithelium by the inner mucin layer.

The lack of stability can be measured by the tear break-up time (TBUT) as the interval of time that elapses between the end of a complete blink and the appearance of the first break in the tear film^3,4^. The first procedure of TBUT, also known as fluorescein BUT (fBUT), was introduced by Norn in 1969^6^, who proposed to instill sodium fluorescein dye in the tears to detect breaks by using a biomicroscope and cobalt blue light. The “magic” number 10 s would indicate the cut off between normal and abnormal tear film^6^. Notwithstanding the clinical fortune of fBUT, which became the most common test for tear film assessment^7–9^, it has been largely recognised for its poor reliability^10,11^, (mainly linked to fluorescein invasiveness)^12^ There have been proposed variations in the fBUT procedure to improve reliability such as a reduction and control of the amount of sodium fluorescein used^13–15^, or performing multiple measures^16^ in different occasions^17^, etc. However, the best way to measure the stability of the tear film should be to use a non-invasive approach^3,18^ that should avoid altering the the tear film (e.g., by increasing the temperature and/or causing reflex blinking with the illumination systems, instilling fluorescein, forcing blinking, etc.)^3,4^. The non-invasive break-up time (NIBUT) is determined as the interval of time that elapses between the end of a complete blink and the appearance of a discontinuity or break in the image of a mire or a grid pattern (keratometer mire or Placido disc) reflected on the anterior tear film surface^4,19^. This approach gives also the possibility to detect changes in the tear film that anticipate a real break-up: for example, a distortion of the grid patterns can be interpreted as a thinning of the film, which can be exploited to measure the tear thinning time^20^. The NIBUT procedure has become widespread^19,21–23^, and nowadays it has been implemented in modern corneal topography systems^24–33^. In these instruments, NIBUT is measured in an automatic way by algorithms that can assess the break-up from the video acquired by videokeratography^34^. However, differences in Placido disc (e.g., number and size of rings), background illumination, and algorithms might cause differences in results. In this view, the present study, arranged in two experiments, was aimed to evaluate the agreement and repeatability of a recently developed topography-based NIBUT in comparison with two long-standing manual NIBUT procedures and fBUT, as well as the agreement between the NIBUT achieved by the automatic algorithm and a manual assessment.

## Methods

### First experiment: agreement and repeatability of different BUT measurement procedures

#### Participants

To evaluate the sample size needed for the study, a priori analysis was performed by the G*Power software (version 3.1.9.4) on preliminary NIBUT and fBUT data measured with the same instruments and procedures used in this study and achieved at the Research Centre (hereinafter referred to as Lab) where the experiment was carried out. Through distribution data (mean and SD) and correlation between them, an effect size of 0.40 was worked out. Considering the need to verify the difference between the means of two repeated test (NIBUT *vs* fBUT), the analysis type was set on matched pairs t-test (two-sided). Fixing an α error and 1-β (power) at 0.05 and 0.80 respectively, the resulting sample size was N = 41.

Thus, forty-three participants (age: 23.1 ± 2.1 years; range 18.1–29.3 years; sixteen males and twenty-seven females) were enrolled in the study on a voluntary basis. The inclusion criteria are reported in Table 1. Eventual dry eye symptoms were monitored by Ocular Surface Disease Index (OSDI) questionnaire (average score: 10.7 ± 10.0; range: 0.0–39.6).

**Table 1:** Inclusion criteria for subjects enrolled in the study.

Inclusion criteria
Age ≥ 18 years
Non-contact lens wearers
Absence of any known ocular pathology and not being subjected to refractive surgery or ocular drug treatment
Absence of any known general pathology
Not taking any ocular or systemic medication known to affect the ocular surface
Not being in state of pregnancy
Able and willing to adhere to any study instructions and complete all specified evaluation
Read, indicate understanding of, and sign informed consent

All participants gave written informed consent, and all procedures were conformed to the Declaration of Helsinki and were approved by the Board of Optics and Optometry of the University of Milano-Bicocca (February, 11th, 2019).

#### Instruments

**Figure 1:**
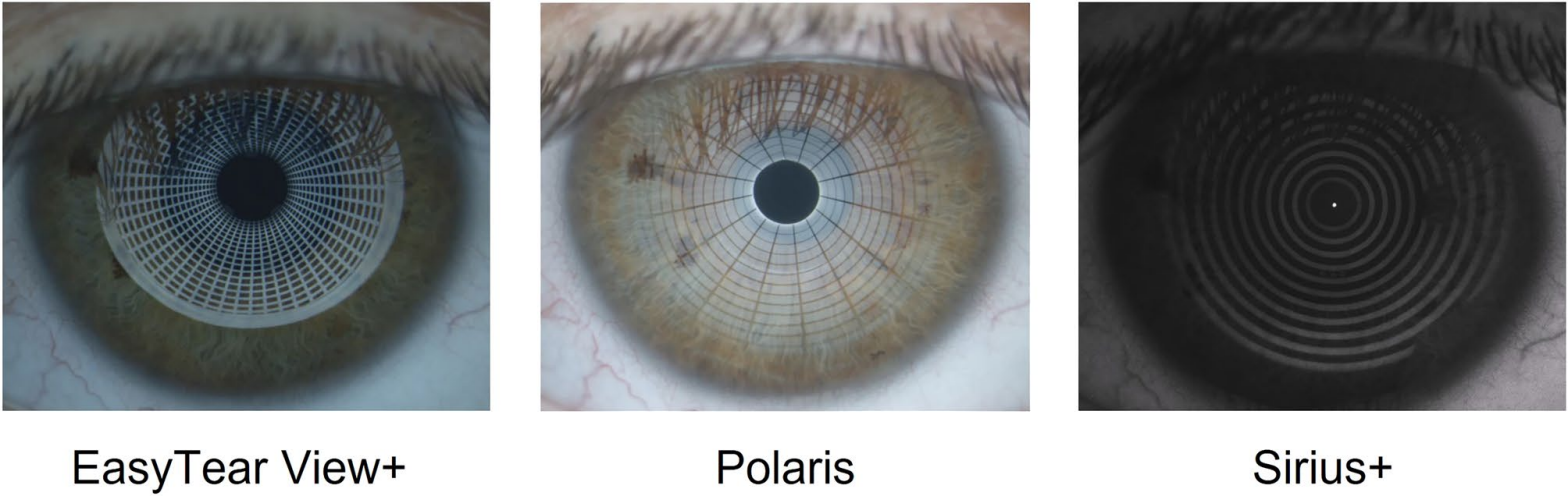
Example on the same subject of the grids reflected by tear film by using EasyTear View + (left), Polaris (centre) and Sirius+ (right).

Three different devices were used to collect NIBUT data. Two devices, the EasyTear View + (Easytear, Rovereto, Italy) and the Polaris (CSO, Florence, Italy), have a similar structure with a cylindrical internal light source and a diffuser that allow to project diffuse cold light (white LED). The insertion of specific grids inside the internal cylinder light source of the instrument allows the projection of concentric rings onto the tear film (Fig. 1), thus the possibility to detect irregularities of the reflected image. Both instruments were mounted on a digital slit lamp (HR Elite, CSO, Florence, Italy) that allows video recording. The third device, the Sirius+ (CSO, Florence, Italy), is a Placido disc topographer integrated with a Scheimpflug tomographer (Fig. 1). The algorithm integrated in the dedicated software (Phoenix v.4.0, CSO, Florence, Italy) splits the Placido disc’s ring projection into a pre-set number of circular sectors (tiles) with the same area. For each sector, the algorithm keeps a trace of the changes (disruption of the projected ring) in each sector’s structure as time passes by. Only changes that persist until the end of the recording are considered as break-up, whereas a change that is restored to its original shape by the end of the recording is considered a false positive due to possible artifacts (e.g. small elements moving into the tear film layer). Disruptions of the projected ring that are visible since the beginning of the recording, such as eyelash shadow, are excluded from the processing. The algorithm can provide the first break-up regardless the sectors or the break-up map; the first break-ups are displayed topographically for each sector.

#### Procedure

**Figure 2:**
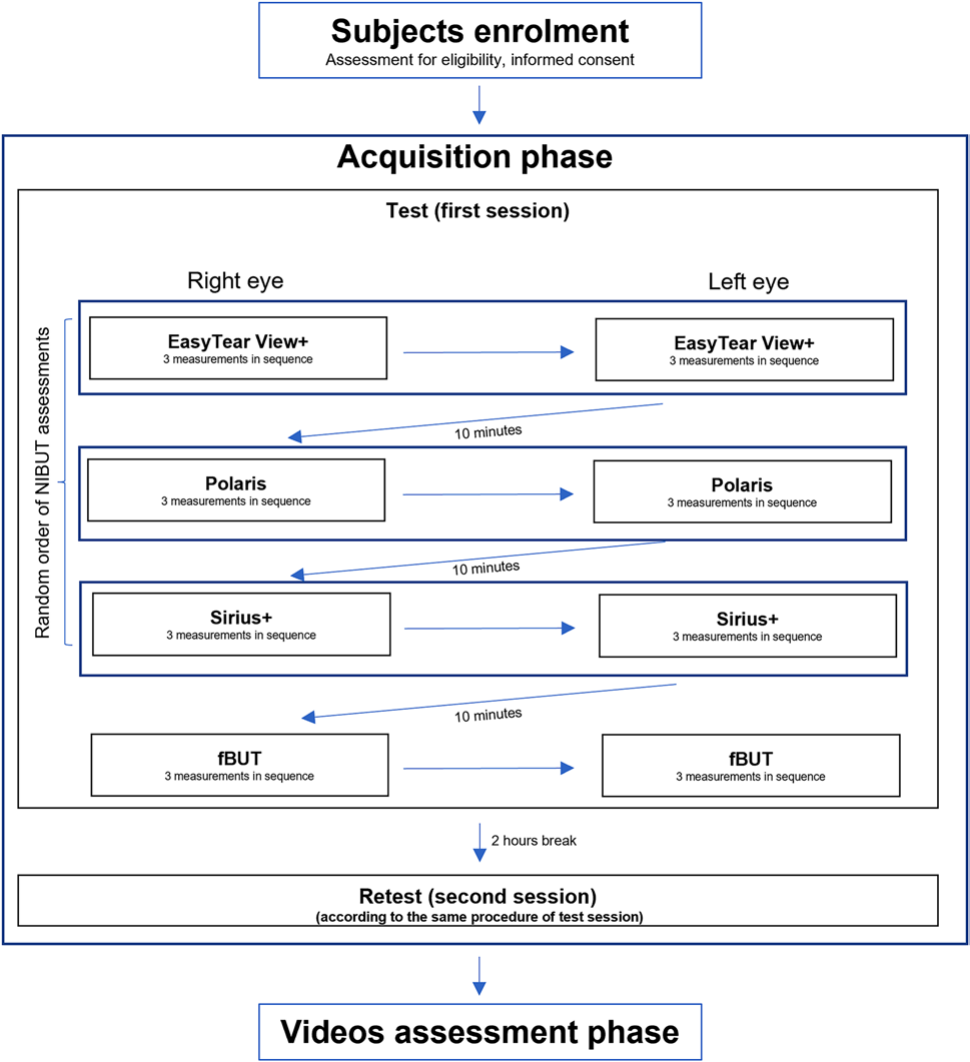
Flow diagram of the study design. After the enrollment, participants run the first set of NIBUT measurements (test), the same series of measurements were retaken (retest) in the same day at least 2 h after the first set of measurements.

All measurements were performed in the same Lab following the procedure reported in Fig. 2. The same researcher performed all NIBUT measurements by employing the three devices in a randomised order, with an interval between the different procedures of minimum 10 min to wash out any potential tear film destabilization due to the previous measurements^35,36^. For each instrument, three NIBUT measurements were achieved in a row for each eye.

EasyTear View+ and Polaris measurements were video recorded with the digital slit lamp. As for the Sirius+, the standard length of NIBUT video recording by the software was extended to 50 s to allow the detection of long break-up times. After the NIBUT measurements, the same researcher performed a standard fBUT three times in a row for each eye. fBUT was carried out always at the end due to its invasiveness compared to the NIBUT measurements. The fBUT was performed by fluorescein sodium strips (I-DEW FLO, Endot, UK) used according to Pult & Riede-Pult procedure^14^, with slit lamp (HR Elite, CSO, Florence, Italy), blue cobalt and yellow filters. The fBUT was video recorded with the digital slit lamp. Subjects, as for the non-invasive devices, were asked to blink twice and then trying to avoid blinking as long as possible. The fBUT was video recorded with the digital slit lamp. Test–retest reliability was evaluated performing the same series of measurements (according to the order randomly selected for each specific subject) in the same day at least 2 h after the first set of measurements.

EasyTear View+ and Polaris NIBUTs, as well as fBUTs, were evaluated on the recorded videos by a researcher that was masked of the other instruments results. The researcher was instructed to play the videos with the task to detect the very first break-up and therefore was given the possibility to rewind the video to better identify the break-up. As far as concerns the Sirius+, only the first NIBUT (first disruption of the projected rings irrespectively to the sector) was obtained directly by the automatic algorithm analysis.

### Data analysis

All the following data analyses were carried out for right and left eye separately^37^. All data sets did not result normally distributed (Shapiro–Wilk test; *p* < 0.005), thus non-parametric statistics were used. The agreement among the four BUT assessment procedures was investigated by Friedman’s test, then a matched comparison (Wilcoxon signed-rank test) was performed between each pair of measurements. Bonferroni adjustment was used to correct for multiple comparisons for post-hoc analyses. Spearman coefficient of correlation was calculated for each pair of measurements too.

Intra-observer repeatability was evaluated with the coefficients of precision (CP), repeatability (CR) and variation (CV). CP was calculated as 1.96 * s_w_ (s_w_ is the within-subjects standard deviation for repeated measures). CR was calculated as $1.96*\sqrt{{S}_{w}^{2}*2}$ that is the value under which it would be the difference between two measurements in the 95% of probability^38^. CV was calculated as s_w_ divided by the overall sample mean.

Test–retest reliability was evaluated for each procedure (mean of the three measures at test and mean of the three measures at retest) by Intraclass Correlation Coefficient (ICC) based on mean measurement, absolute agreement, two-way mixed effects model^39^. The 95% confidence interval was calculated. Reliability is considered slight, fair, moderate, substantial and excellent if ICC is comprised between 0.01 and 0.20, 0.21 and 0.40, 0.41 and 0.60, 0.61 and 0.80, and more than 0.80 respectively^40^. A comparison between test and retest was also performed by matched-pairs Wilcoxon test. The statistical analyses were performed with SPSS version 2.8 (IBM SPSS Statistics, USA).

### Second experiment: agreement between manual and automatic NIBUT measured by Sirius+

#### Sample

The present part of the study did not require a direct enrollment of participants and no ethical issue; therefore, the effect size of the experiment was determined using a post hoc procedure by the G*Power software (G*Power; version 3.1.9.4) for a comparison between means of two distributions by Wilcoxon test. Through distribution data (mean and SD) of automatic and manual NIBUT (first and overall measures) and correlation between them, with a sample size of N = 85, the effect size was worked out. Fixing an α error at 0.05, the power effect (1-β) resulted of 0.97 and 0.60 for the difference between the mean of the automatic measure with the first manual NIBUTs (both observers) and the overall mean of all manual NIBUTs (both observers) respectively.

Thus, eighty-five videos of the NIBUT procedure previously performed with Sirius+ (CSO, Florence, Italy) were selected according to the following criteria:

- No blinking during the length of the recoding
- The first break-up, detected by automatic assessment, should occur before 17 s (limiting the study to length compatible with tear film instability in which information about the difference between manual and automatic assessment is more useful)
- No areas grossly out of focus
- No missing fixation (due to movements of the eye or head)
- No gross irregularities of the tear film (e.g., mucus, air bubbles, etc.).

#### Procedure

**Figure 3:**
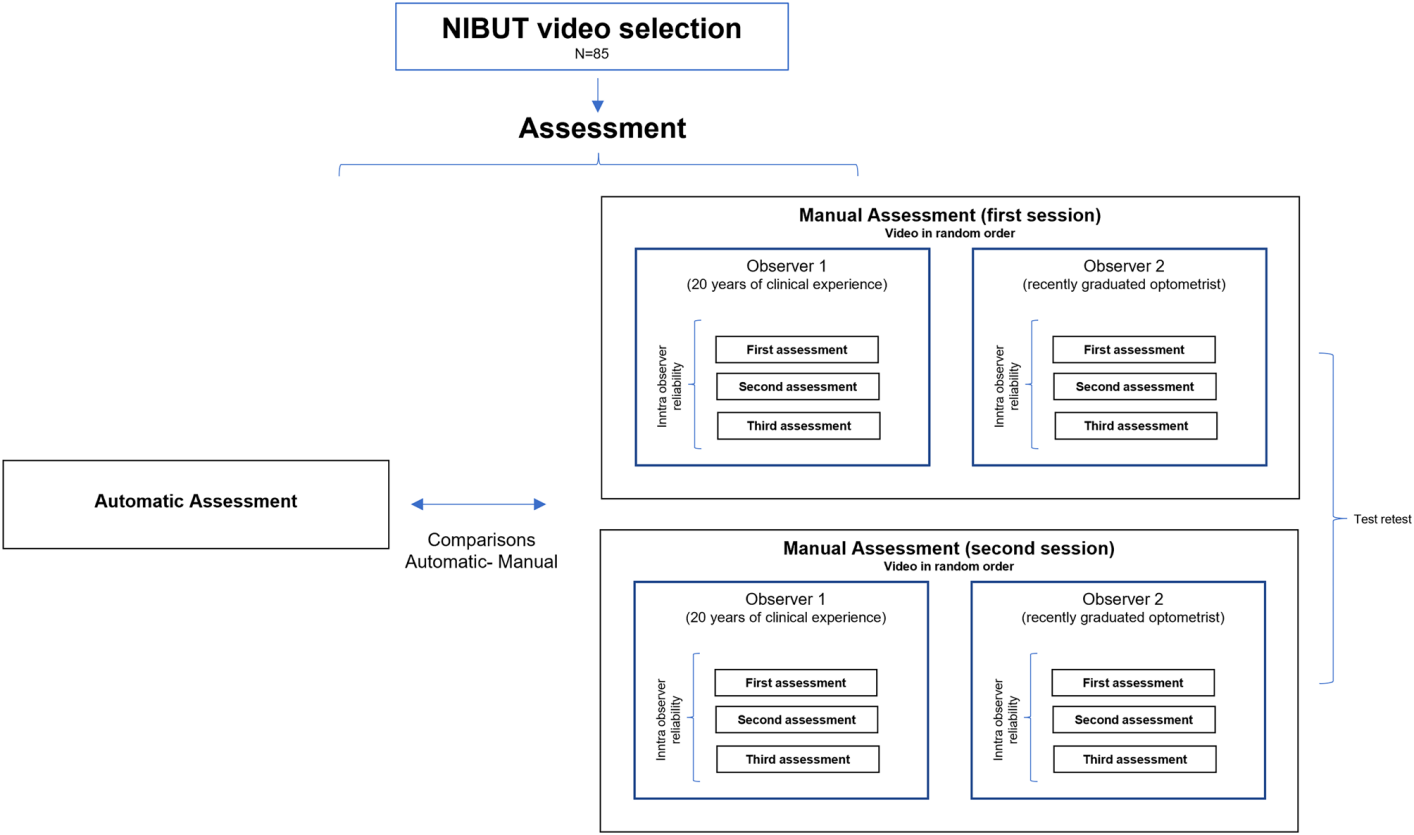
Flow diagram of the study design adopted in the second experiment. After videos’ selection, the two observers assessed the videos separately in random order three times (first session). The assessment was repeated (retest) after 15 days.

A flow diagram of the study design is represented in Fig. 3. Two observers with different clinical experience were chosen to evaluate the videos and investigate a possible influence of the experience on the manual (subjective) assessment of NIBUT. Observer 1 was a researcher and an eye care practitioner with more than 20 years of clinical experience. Observer 2 was a recently graduated optometrist with less than one year of experience in clinical practice. The two observers assessed each single video (played in freeware software on the same laptop) in random order, measuring the NIBUT three times in a row (first session). Before proceeding with the evaluation of the videos, common instructions on what should be identified as 'break-up' were provided to both observers. They were required to play the video and stop it as soon as the first break-up (discontinuity or break in the image of the rings) appears; the break-up time was recorded, and the video was rewound from the beginning to perform the other two measures. Observers repeated the assessment after 15 days (second session). The 85 videos were provided in random order (different from the one used in the first session) and without any information about the measures determined during the first session.

The same 85 videos were analysed by the automatic algorithm, the two observers were masked of the instrument results.

### Data analysis

All data (first break-up time) used to assess the agreement between manual and automatic NIBUT measured by Sirius+ did not result normally distributed (Shapiro–Wilk test; *p* < 0.005).

Comparison between automatic (first break-up time) and manual measurements was performed by Wilcoxon test and Spearman correlation on the first manual measurement (mean of the first measure at the test session by the two observers), on the overall manual measurement (mean of all manual measures in both sessions) and on the mean manual measurement separately for each observer. The same statistical tests were used to compare manual NIBUT of the two observers. Bonferroni adjustment was used to correct for multiple comparisons for post-hoc analyses. Spearman coefficient of correlation was calculated for each pair of measurements too. Friedman ANOVA for repeated measures was used to evaluated differences in the three NIBUT assessments performed by two observers in the two sessions.

Intra-operator repeatability was calculated for each of the two observers using the same coefficients previously described in data analysis of the first experiment.

Test–retest reliability (between the two sessions) was evaluated for each observer (mean of the three measures at test and mean of the three measures at retest) by ICC^39^, as aforementioned in data analyses of the first experiment, and by matched-pairs Wilcoxon test.

## Results

### First experiment: agreement and repeatability of different BUT measurement procedures

**Figure 4:**
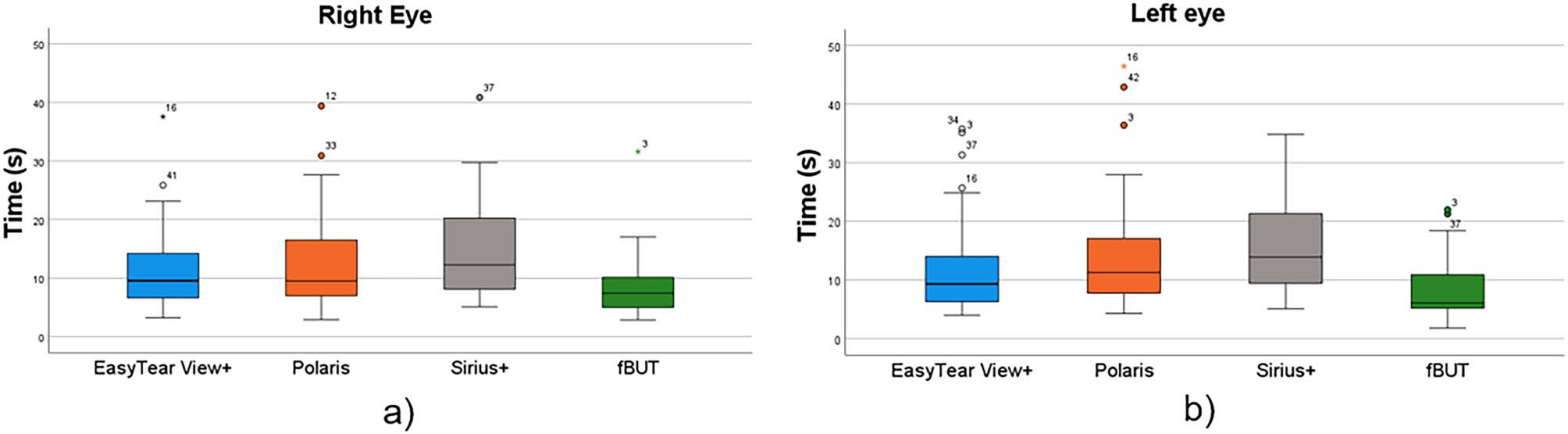
Box and whisker plot of the BUT distribution with the four procedures in right eye (**a**) and left eye (**b**). A significant difference was found among the four measures in both eyes (Friedman ANOVA; *p* < 0.001).

On the right eye, BUT (average of test and retest ± SD) resulted 12.0 ± 7.6, 12.8 ± 6.8, 14.8 ± 8.0, and 8.7 ± 5.2 s with the EasyTear View+, Polaris, Sirius+, and fluorescein-based procedure, respectively (Fig. 4a). On the left eye, BUT resulted 12.0 ± 8.2, 14.1 ± 9.8, 15.6 ± 7.8, and 8.6 ± 5.0 s with the EasyTear View+, Polaris, Sirius+, and fluorescein-based procedure, respectively (Fig. 4b). Friedmann’s analysis of variance showed a significant difference between the four measures in both eyes (*p* < 0.001). Post-hoc testing among the four procedures is reported in Table 2 along with correlations. All paired comparisons with fBUT showed significant difference for both eyes. Conversely, all paired comparisons between NIBUT procedures on the right eye were not significantly different, whereas on the left eye the comparisons between EasyTear View+, and the other two NIBUT procedures (Polaris and Sirius+) were significant, but the comparison between Polaris and Sirius+ was not. All correlations among procedures resulted significant (*p* < 0.001).

**Table 2:** Paired comparisons (Wilcoxon test) and correlation (Spearman Rho) among the single four procedures in the two eyes.

Procedure	Right eye			Left eye		
	EasyTear View+	Polaris	Sirius+	EasyTear View+	Polaris	Sirius+
Polaris	Wilcoxon; *P* = 0.14			Wilcoxon; *P* = 0.006*		
	Spearman Rho = 0.68 (*p* < 0.001)*			Spearman Rho = 0.87 (*p* < 0.001)*		
Sirius+	Wilcoxon; P = 0.022	Wilcoxon; P = 0.016		Wilcoxon; *P* = 0.002*	Wilcoxon; *P* = 0.07	
	Spearman Rho = 0.43 (*p* < 0.005)*	Spearman rho = 0.63 (*p* < 0.001)*		Spearman Rho = 0.56 (*p* < 0.001)*	Spearman Rho = 0.47 (*p* < 0.001)*	
fBUT	Wilcoxon; *P* < 0.001*	Wilcoxon; *P* < 0.001*	Wilcoxon; *P* < 0.001*	Wilcoxon; *P* < 0.001*	Wilcoxon; *P* < 0.001*	Wilcoxon; *P* < 0.001*
	Spearman Rho = 0.59 (*p* < 0.001)*	Spearman Rho = 0.70 (*p* < 0.001)*	Spearman Rho = 0.53 (*p* < 0.001)*	Spearman Rho = 0.77 (*p* < 0.001)*	Spearman Rho = 0.71 (*p* < 0.001)*	Spearman Rho = 0.49 (*p* < 0.001)*

*Significant comparisons (after Bonferroni correction for multiple comparisons, alpha was lowered to 0.008) and significant correlations are reported in bold.

**Figure 5:**
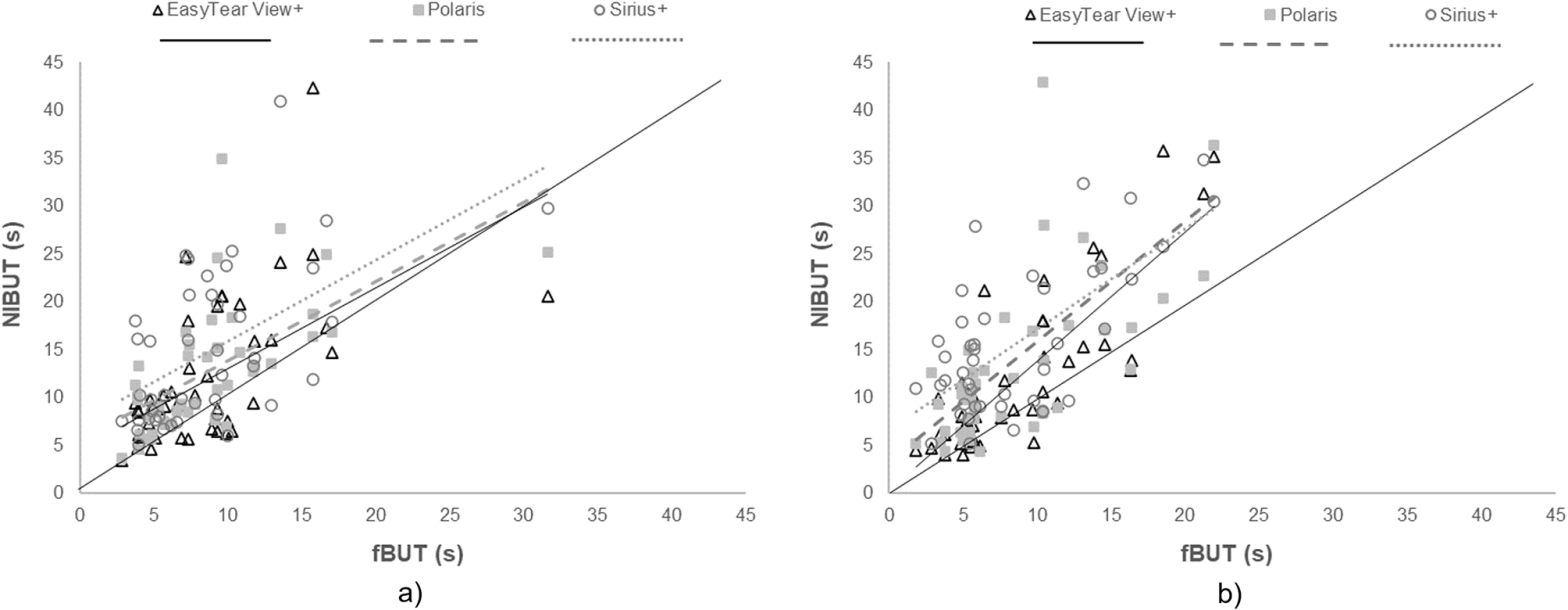
Scatterplot of the BUTs between fBUT and the three NIBUTs in right eye (**a**) and left eye (**b**).

To investigate the relationship between invasive and non-invasive procedure, fBUT values were reported as a function of the three NIBUTs (Fig. 5).

Intra-observer repeatability for the four instruments, in the two sessions, was rather poor as it is possible to see from the high values of CP, CR, and CV reported in Table 3.

**Table 3:** Coefficient of precision (CP), coefficient of repeatability (CR) and coefficient of variation (CV) for the measures with the four instrument/procedure in the first session (test) and in second session (retest).

	Right eye				Left eye			
	EasyTear View+	Polaris	Sirius+	fBUT	EasyTear View+	Polaris	Sirius+	fBUT
Test	CP = 13.8 s	CP = 10.6 s	CP = 13.8 s	CP = 7.1 s	CP = 10.6 s	CP = 9.5 s	CP = 13.9 s	CP = 8.6 s
	CR = 19.5 s	CR = 15.0 s	CR = 19.6 s	CR = 10.0 s	CR = 15.1 s	CR = 13.4 s	CR = 19.7 s	CR = 12.2 s
	CV = 0.56	CV = 0.42	CV = 0.45	CV = 0.41	CV = 0.42	CV = 0.35	CV = 0.45	CV = 0.47
Retest	CP = 7.6 s	CP = 9.0 s	CP = 14.3 s	CP = 6.1 s	CP = 9.0 s	CP = 11.1 s	CP = 12.4 s	CP = 4.5 s
	CR = 10.8 s	CR = 12.7 s	CR = 20.3 s	CR = 8.6 s	CR = 12.7 s	CR = 15.7 s	CR = 17.5 s	CR = 6.4 s
	CV = 0.34	CV = 0.36	CV = 0.53	CV = 0.36	CV = 0.42	CV = 0.39	CV = 0.41	CV = 0.30

The results of test–retest are shown in Table 4 that reports the descriptive statistics of BUT, ICC, and p-values of paired comparison. ICC was substantial (between 0.61 and 0.80) for the EasyTear View+ measures on both eyes, for the Polaris in left eye, for the Sirius+ and the fBUT in the right eye. For the Sirius+ and the fBUT on the left eye the ICC was moderate and for the Polaris on the right eye was fair^40^. No test–retest difference was found for all procedures. Moreover, Bland–Altman plots of the test–retest measurements indicate a good agreement between the first and second measurement without any proportional bias (see Supplementary Figs. S1 and S2 online): all correlations (Spearman Rho) between the mean of test and retest and the difference retest-test were not significant.

**Table 4:** Test–Retest (N = 43). Descriptive statistics of BUT (s), Intraclass Correlation Coefficient (ICC) between test and retest measures calculated with two-way mixed effects model, absolute agreement, mean measurement (*p < 0.05; **p < 0.01; ***p < 0.001), and *p* values of paired comparison between test and retest (Wilcoxon test).

Procedure	Right eye				Left eye			
	Test (s) Mean ± SD; Median	Retest (s) Mean ± SD; Median	ICC and 95% confidence intervals	Comparison (*p* value of Wilcoxon-test)	Test (s) Mean ± SD; Median	Retest (s) Mean ± SD; Median	ICC and 95% confidence intervals	Comparison (*p* value of Wilcoxon test)
EasyTear View+	12.7 ± 9.6; 9.4	11.3 ± 7.1; 9.6	0.76*** (0.56–0.87)	*P* = 0.52	13.1 ± 10.4; 8.4	10.9 ± 7.7; 7.9	0.76*** (0.55–0.87)	*P* = 0.12
Polaris	13.0 ± 9.3; 10.2	12.7 ± 8.1; 9.5	0.37 (− 0.18–0.66)	*P* = 0.24	13.7 ± 10.7; 9.4	14.5 ± 10.7; 9.3	0.81*** (0.64–0.90)	*P* = 0.50
Sirius+	15.8 ± 9.5; 13.2	13.8 ± 8.1; 10.8	0.78*** (0.59–0.88)	*P* = 0.08	15.9 ± 9.7; 11.8	15.4 ± 9.1; 13.2	0.57** (0.56–0.87)	*P* = 0.90
fBUT	8.9 ± 6.0; 6.8	8.6 ± 6.2; 7.0	0.61** (0.28–0.79)	*P* = 0.38	9.4 ± 7.5; 6.7	7.8 ± 4.1; 6.3	0.55** (0.19–0.76)	*P* = 0.74

**Figure 6:**
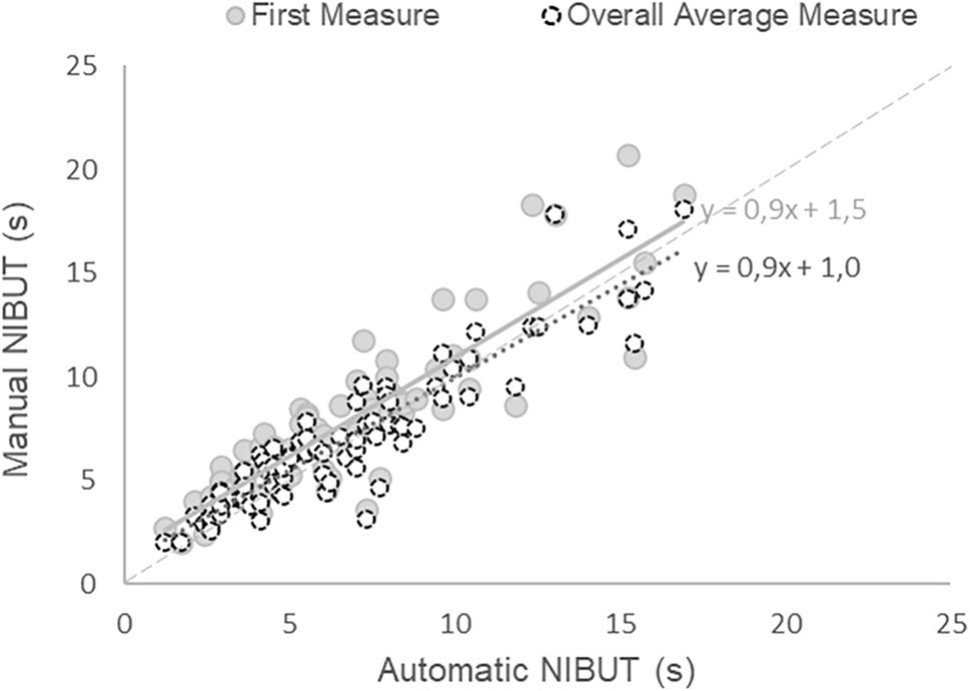
Scatterplot between the automatic NIBUT and the two manual NIBUTs analysed: first measure (grey circles and continuous grey regression line) and overall average (dotted circles and dotted black regression line).

### Second experiment: agreement between manual and automatic NIBUT measured by Sirius+

The distribution of the automatic NIBUTs resulted (mean ± SD) 6.6 ± 3.6 s (range 1.2–16.9 s). The manual NIBUT was (mean ± SD) 7.7 ± 3.8 s (range 2.0–20.7 s) and 6.9 ± 3.5 s (range 2.0–18.1 s) for the first measurement (only first session) and the overall measurement, respectively. A statistically significant difference was found between the automatic NIBUT and both the first manual and the average manual measurement (Wilcoxon test; *p* < 0.001). Figure 6 shows the scatterplot between the automatic NIBUT and two manual NIBUTs (first and overall average). Pearson correlation coefficient calculated between automatic and manual NIBUTs resulted 0.89 (*p* < 0.001), and 0.90 (*p* < 0.001) for the first and the overall and the overall average manual measurements respectively.

NIBUT data achieved by the two observers in the two sessions are reported in Table 5 along with pair comparisons between the two observers for each measure, and pair comparisons between each manual NIBUT achieved by each observer and automatic NIBUT. All NIBUTs resulted significantly different between the two observers, but all were significantly correlated (all Pearsons correlations resulted higher than 0.85; *p* < 0.001). Friedman ANOVA for repeated measures showed a reduction in manual NIBUT in the 3 measurements in a row both for observer 1 (*p* = 0.03) and observer 2 (*p* < 0.001) in the first session, as well as in the second session (*p* < 0.001 for both Observers). All manual NIBUTs measured by observer 1 (except the second and third measures in the second session), resulted significantly longer than automatic NIBUT (between 0.3 and 1.6 s), whereas for observer 2 the difference was significant only for the first NIBUT in first session (longer time), the second, third NIBUT in the second session and the average NIBUT in the second session (shorter time). However, all manual NIBUTs achieved by the two observers and the automatic NIBUT resulted strongly correlated (all Spearman Rho higher than 0.83; *p* < 0.001).

**Table 5:** Descriptive statistics (Mean ± SD and range) of NIBUT (s) manually measured by the two observers (Obs1 and Obs 2) in the two sessions (N = 85). Paired comparisons between observers for each manual measure (Wilcoxon test in fifth row) and correlation (sixth row), as well as paired comparisons between automatic and each manual measure achieved by the two observers (Wilcoxon test, tenth and eleventh row) and correlation (Spearman Rho; twelfth and thirteenth row) are also reported.

	First session (Test)				Second session (Retest)				Overall average manual Mean ± SD (Range) (s)
	First manual Mean ± SD (Range) (s)	Second manual Mean ± SD (Range) (s)	Third manual Mean ± SD (Range) (s)	Average Mean ± SD (Range) (s)	First manual Mean ± SD (Range) (s)	Second manual Mean ± SD (Range) (s)	Third manual Mean ± SD (Range) (s)	Average Mean ± SD (Range) (s)	
Observer 1 (Obs 1)	8.2 ± 4.2 (1.9–22.7)	7.9 ± 3.9 (1.9–20.9)	7.7 ± 3.7 (1.9–19.0)	7.9 ± 3.9 (2.0–20.4)	7.3 ± 3.6 (2.0–19.2)	6.9 ± 3.5 (1.8–18.3)	6.8 ± 3.4 (2.2–18.7)	7.0 ± 3.5 (2.0–18.4)	7.5 ± 3.6 (2.0–19.0)
Observer 2 (Obs 2)	7.2 ± 3.7 (1.2–19.7)	6.5 ± 3.6 (1.5–18.6)	6.3 ± 3.4 (1.6–17.5)	6.7 ± 3.5 (1.7–17.7)	6.2 ± 3.6 (1.4–19.3)	5.8 ± 3.4 (1.0–17.1)	5.8 ± 3.3 (1.2–18.2)	6.0 ± 3.4 (1.2–17.4)	6.3 ± 3.4 (1.7–17.3)
Comparison Obs 1—Obs 2 (Wicoxon test)	*p < 0.001	*p < 0.001	*p < 0.001	*p < 0.001	*p < 0.001	*p < 0.001	*p < 0.001	*p < 0.001	*p < 0.001
Correlation Obs1—Obs 2 (Sperman Rho)	*0.86 (*p* < 0.001)	*0.85 (*p* < 0.001)	*0.87 (*p* < 0.001)	*0.89 (*p* < 0.001)	*0.88 (*p* < 0.001)	*0.90 (*p* < 0.001)	*0.93 (*p* < 0.001)	*0.92 (*p* < 0.001)	*0.94 (*p* < 0.001)
Difference between Automatic and Manual NIBUT Obs 1 (s)	− 1.6	− 1.3	− 1.2	− 1.4	− 0.8	− 0.3	− 0.3	− 0.5	− 0.9
Difference between Automatic and Manual NIBUT Obs 2 (s)	− 0.6	0.1	0.3	-0.1	0.4	0.7	0.7	0.6	0.3
Comparison Automatic-Obs 1 (Wicoxon test)	*p < 0.001	*p < 0.001	*p < 0.001	*p < 0.001	*p < 0.001	*p* = 0.03	*p* = 0.04	*p < 0.001	*p < 0.001
Comparison Automatic-Obs 2 (Wicoxon test)	*p = 0.003	*p* = 0.94	*p* = 0.27	*p* = 0.40	*p* = 0.07	*p < 0.001	*p < 0.001	*p < 0.001	*p* = 0.33
Correlation Automatic—Obs 1 (Sperman Rho)	*0.87 (*p* < 0.001)	*0.88 (*p* < 0.001)	*0.88 (*p* < 0.001)	*0.89 (*p* < 0.001)	*0.84 (*p* < 0.001)	*0.86 (*p* < 0.001)	*0.87 (*p* < 0.001)	*0.87 (*p* < 0.001)	*0.89 (*p* < 0.001)
Correlation Automatic—Obs 2 (Sperman Rho)	*0.84 (*p* < 0.001)	*0.84 (*p* < 0.001)	*0.85 (*p* < 0.001)	*0.86 (*p* < 0.001)	*0.83 (*p* < 0.001)	*0.87 (*p* < 0.001)	*0.88 (*p* < 0.001)	*0.87 (*p* < 0.001)	*0.89 (*p* < 0.001)

*Significant comparisons (after Bonferroni correction for multiple comparisons, alpha was lowered to 0.017) and significant correlations are reported in bold.

Table 6 shows the statistical coefficients of intra-operator repeatability (among the three measures performed in a row in each session), separately for the two observers in the two sessions. Coefficients show good intra-operator repeatability in both observers.

**Table 6:** Coefficient of precision (CP), coefficient of repeatability (CR) and coefficient of variation for the manual measures of NIBUT performed by observer 1 and observer 2 in the first session (test) and in second session (retest).

	Observer 1	Observer 2
Test	CP = 1.68 s; CR = 2.37 s; CV = 0.11	CP = 1.71 s; CR = 2.41 s; CV = 0.13
Retest	CP = 1.31 s; CR = 1.85 s; CV = 0.10	CP = 1.32 s; CR = 1.87 s; CV = 0.11

Table 7 reports the descriptive statistics of manual NIBUTs achieved by the two observers and their average at test and retest, the ICC between test and retest measures, and p values of paired comparison between test and retest (Wilcoxon test). ICC was excellent (over than 0.80)^40^ for both observers. However, NIBUTs at retest resulted significantly shorter than test for both observers (*p* < 0.001). Finally, Bland–Altman plots of the test–retest measurements (see Supplementary Fig. 3S online) show a proportional bias for observer 1 (Spearman Rho = − 0.29; *p* = 0.008), indicating that the longer the NIBUT the shorter the retest compared to test. No proportional bias was found for the observer 2 (Spearman Rho = − 0.04; *p* = 0.74).

**Table 7:** Test–Retest (N = 85). Descriptive statistics of NIBUT (sec), Intraclass Correlation Coefficient (ICC) between test and retest measures calculated with two-way mixed effects model, absolute agreement, mean measurement (* *p* < 0.05; **p < 0.01; ***p < 0.001), and *p* values of paired comparison between test and retest (Wilcoxon test).

Procedure	Test Mean ± SD; (Range) (s)	Retest (s) Mean ± SD; (Range) (s)	ICC and 95% confidence intervals	Comparison (*p* value of Wilcoxon test)
Observer 1 (Mean of three measurements)	7.9 ± 3.9; (2.0–20.4)	7.0 ± 3.5; (2.0–18.4)	0.95*** (0.84–0.98)	*P* < 0.001
Observer 2 (Mean of three measurements)	6.7 ± 3.5; (1.7–17.7)	6.0 ± 3.4; (1.2–17.4)	0.95*** (0.90–0.98)	*P* < 0.001
Average of observers	7.3 ± 3.6; (1.9–18.7)	6.5 ± 3.4; (1.6–17.6)	0.97*** (0.82–0.99)	*P* < 0.001

## Discussion

Two different experiments were carried out to evaluate the NIBUT assessment of Sirius+, a recently developed Placido-based topographer integrated with a Scheimpflug tomographer. Even though its clinical application has been already reported in the literature^41–44^, no data about its level of agreement with other devices/procedures, and repeatability is available. To clarify the discussion of the results obtained in the two experiments, the outcomes have been divided into specific paragraphs.

### Agreement between NIBUT procedures and fBUT

The first part of the study showed that NIBUT was longer than fBUT, independently from the device employed, and this result is in agreement with the literature^12,15,45–47^. However, elsewhere in literature automatic NIBUT was also found to be shorter than fBUT^27,48^. It has been proposed that the shorter fBUT might be induced by the instillation of fluorescein which would reduce the stability of the tear film^15,19^. When the amount of instilled fluorescein is reduced, the difference between NIBUT and fBUT decreases^15^. However, it has also been found that increasing the delivered volume of fluorescein solution by the glass rod technique (micropipette) lengthened fBUT^47,49^. In a recent paper, NIBUT measurements were carried out with Sirius+ without and with fluorescein that caused a prolongation in the NIBUT, labelled as “de-naturation” of the tear film^50^.

In the present study, a caveat of the difference between fBUT and NIBUTs might be the fact that the sequence of the measurements was not fully randomised: due to its invasiveness fBUT was carried out always at the end. Despite washout intervals, this practice may have contributed to decreased tear film stability, increasing the difference between fBUT and NIBUTs.

Furthermore, another source of shorter times with fBUT might be the different area covered by fBUT and NIBUT assessments. In many participants, the shadow of the lashes on the superior area of the Placido rings (Fig. 1) made the measurement impossible in this area for both the manual and the automated assessment of NIBUT procedures. Moreover, the Placido rings were reflected only in a reduced area of the cornea (Fig. 1). This made the area covered by the fBUT procedure larger than the NIBUT procedure, then with the fBUT procedure, it was possible to detect breaks in zones not covered by NIBUT procedures.

### Agreement between NIBUT procedures

Looking at the NIBUT procedures, the first thing to highlight is that the subjective assessment of NIBUT of the EasyTear View+ and the Polaris are extremely close to the findings of Bandlitz et al.^30^ (12.2 ± 6.6 s and 12.0 ± 6.4 s, respectively), who collected data with the same paradigm (two sessions in the same day) on individuals with very similar age (24.2 ± 3.6 years *vs* 23.1 ± 2.1 years in this work). The present study showed no difference between Polaris and Sirius+ in both eyes and between EasyTear View+ and Sirius+ in the right eye. However, few comparisons displayed a statistical difference (see Table 2). This result is not clear to interpret. Considering that the three NIBUT procedures are non-invasive and based on a “concentric ring grid”, the results might be expected to be similar, as reported for four NIBUT devices (EasyTear View+ , Keratograph 5 M, Polaris, and Tearscope Plus)^30^. However, other studies evidenced a poor agreement between different NIBUT procedures^51,52^. Furthermore, NIBUT values in healthy population, measured by grids or Placido discs, have shown extreme variability, ranged between 10 and 50 s^19,22,23^. Therefore, it should be considered that many factors could induce variability, such as different age and ethnicity of the subjects assessed, the various sizes, brightness and coverage (e.g., due to corneal curvature) of Placido discs^21^, and the fact that for some instruments it is still requested a manual (subjective) judgment^46^. Earlier studies comparing automatic and manual NIBUTs consistently found differences^28,31^, but variations in instrument features rather than the detection method (automatic *vs* manual) may have contributed to these differences. For example, the comparison between automated software to achieve a NIBUT by a topographer (Keratograph) and a manual NIBUT performed by Keeler Tearscope showed a shorter time with the former^28^. Also Markulli et al. found that NIBUT of healthy people was significantly greater with the Tearscope-Plus (15.9 ± 10.7 s) than NIBUT achieved with Oculus Keratograph 5 M (8.2 ± 3.5 s)^31^. These results might be because these releases of the software were extremely sensitive to minimal changes in the projected rings (deformation). As for the difference between the two eyes (no difference in right eye among the 3 procedures, and differences limited to EasyTear View+ and the two other NIBUTs in left eye) the only difference in the procedure that might have caused a bias is the missing randomised order between the two eyes (right always first; see Fig. 2). However, this is a simple association with no clear meaningful cause-effect reason.

### NIBUT and fBUT reliability

The first experiment provided information also about the intra-observer repeatability of the measurements. All the procedures showed high values of CP, CR, and CV in both eyes and the test and the retest session (Table 3). fBUT values resulted slightly lower, especially at the retest. The intrasession repeatability of another topography-based NIBUT (VX120 + system) was recently investigated^53^, revealing a within-subject standard deviation (S_w_) of three consecutive measurements of 0.86. This value is notably smaller (from 3.5 to 8 times), than the S_w_ values derived from the coefficients of Table 3 for the four procedures investigated in the present study. However, it is possible that the low Sw value in Molina-Martìn et al.’s study^53^ might be biased by the extremely narrow range of NIBUT in the sample, which is anomalous for healthy subjects. The repeatability coefficients achieved in the present study values might indicate poor reliability of the four procedures, but they could also be linked to the intrinsic very high variability of the phenomenon.

This second hypothesis seems to find a confirmation looking at the values of the repeatability in the second experiment, where the NIBUT is performed on the same video (so the variability of the tear film is nonexistent, and only the variability of the measurement by the observer remains). Finally, the test–retest reliability appeared good for Sirius+ and also for the other two NIBUT procedures (Table 4) in agreement to the results of Bandlitz et al.^30^.

### Agreement between manual and automatic NIBUT performed by Sirius+

Despite the strong correlation between automatic and manual NIBUTs (first and overall average), automatic assessment always provided shorter values than manual measures (of about 1.1 and 0.3 s for the first manual assessment and the average of all assessments, respectively). The difference of about 1 s between the two comparisons depends on the shortening of the manual NIBUT with the repeated measures. This reduction is likely due to the observer’s awareness of the point where the break-up occurs, which affects the following measures. In the first manual measurement, the observer assessed the keratoscopic disc’s projection without any information on the break-up location. Nonetheless, in the subsequent measurements, the observer was allowed to scan more specifically the area where the break-up was previously spotted, then potentially shortening the time. This outcome is something to consider because in clinical practice also the manual NIBUT is quickly transforming for the rapid diffusion of systems that can make a video of the projection of a grid (systems that can be mounted on a video slit lamp such as the Polaris or the EasyTear View+, etc.), allowing the observer to subjectively evaluate offline the recording several times.

However, it can be highlighted that the differences of 1.1 and 0.3 s are clinically negligible (the statistical significance was certainly linked to the big sample studied). Unfortunately, to the best of our knowledge, there is no previous research that compared automatic *vs* manual assessment of NIBUT in the same event (video of the same projection). More often, comparisons between automatic and manual assessment have been carried out on the same subjects, but in different moments and using different instruments^28,31^. Therefore, it is not possible to compare the results of the present study to other studies.

The second experiment showed also a difference between the two observers. One possible explanation might be the different clinical experience of the two observers. The more experienced observer produced longer measures. This might depend on a more prudent judgment that brings the more experienced observer to get the time only when a break-up happened (therefore a discontinuation and no distortion of rings) or a better sensitivity to the break-up of the less experienced observer.

In terms of reliability, the coefficients (Table 7) are very good for both observers. It is interesting to notice that in this case the coefficients are much better (lower values) than the one achieved for manual assessment in the first experiment (Table 4). As already reported in the discussion, this seems to demonstrate that the poor reliability achieved in the NIBUT procedures performed in the first experiment is likely due to the intrinsic variability of the tear film stability. When the phenomenon is the same (second experiment) the intra-observer variability is almost negligible. Also, the test–retest reliability for both the observers is extremely good confirming that the manual assessment of NIBUT is reliable.

The two experiments of the study present some limitations. In the first experiment, the participants were mainly university students, therefore the age was young with a narrow SD. Moreover, the participants were healthy subjects, not selected for DED, although the NIBUT range as well as the variability of symptoms (OSDI score range varied between 0 and 39.6) resulted quite wide.

## Conclusions

The effort to engineer NIBUT devices able to perform an automated measure of tear film stability has made available many different commercialised instruments. However, every “new entry” such as the one investigated in this study, is not interchangeable with other NIBUT devices as well as fluorescein-based procedures. Likely, the difficulty in finding a good level of agreement and repeatability among the several NIBUT devices might be represented by the tear film itself which is a complex system, difficult to model and measure. However, the automated algorithm that measures the NIBUT in the device here studied provided clinically negligible differences from the manual measures achieved on the same video of Placido disc projection.

### Supplementary Information

Supplementary Figures.

## Data Availability

The datasets generated during and/or analysed during the current study are available from the corresponding author on reasonable request.
