# Supplementary material for: Comparing automated and manual assessments of tear break-up time using different non-invasive devices and a fluorescein procedure
Source: Sci Rep. 2024 Jan 30;14:2516. doi: 10.1038/s41598-024-52686-0 (PMC10827797; doi:10.1038/s41598-024-52686-0)
Supplement: Supplementary file 1 — Supplementary Figures. [file 41598_2024_52686_MOESM1_ESM.docx]

**Supplementary Information**


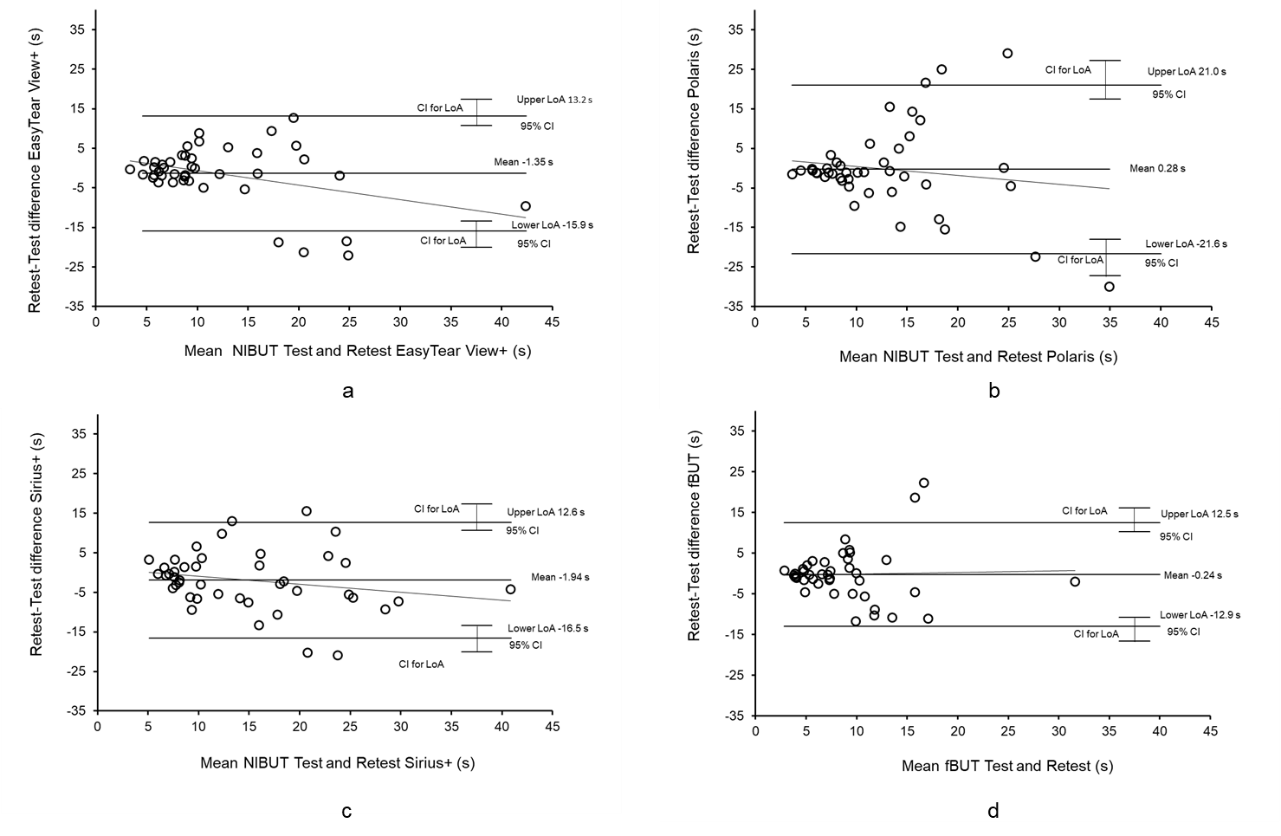


Figure S1: Bland–Altman plot for the test–retest for the four measurements in right eye in the first experiment of the study. The limits of agreement were calculated as mean ± 1.96 SD. Spearman Rho (correlation between the mean of test-retest and the difference between retest-test) resulted -0.07, -0.05, -0.25 and -0.15 (all n.s) for EasyTear View+ (a), Polaris (b), Sirius+ (c), and fBUT (d) respectively.


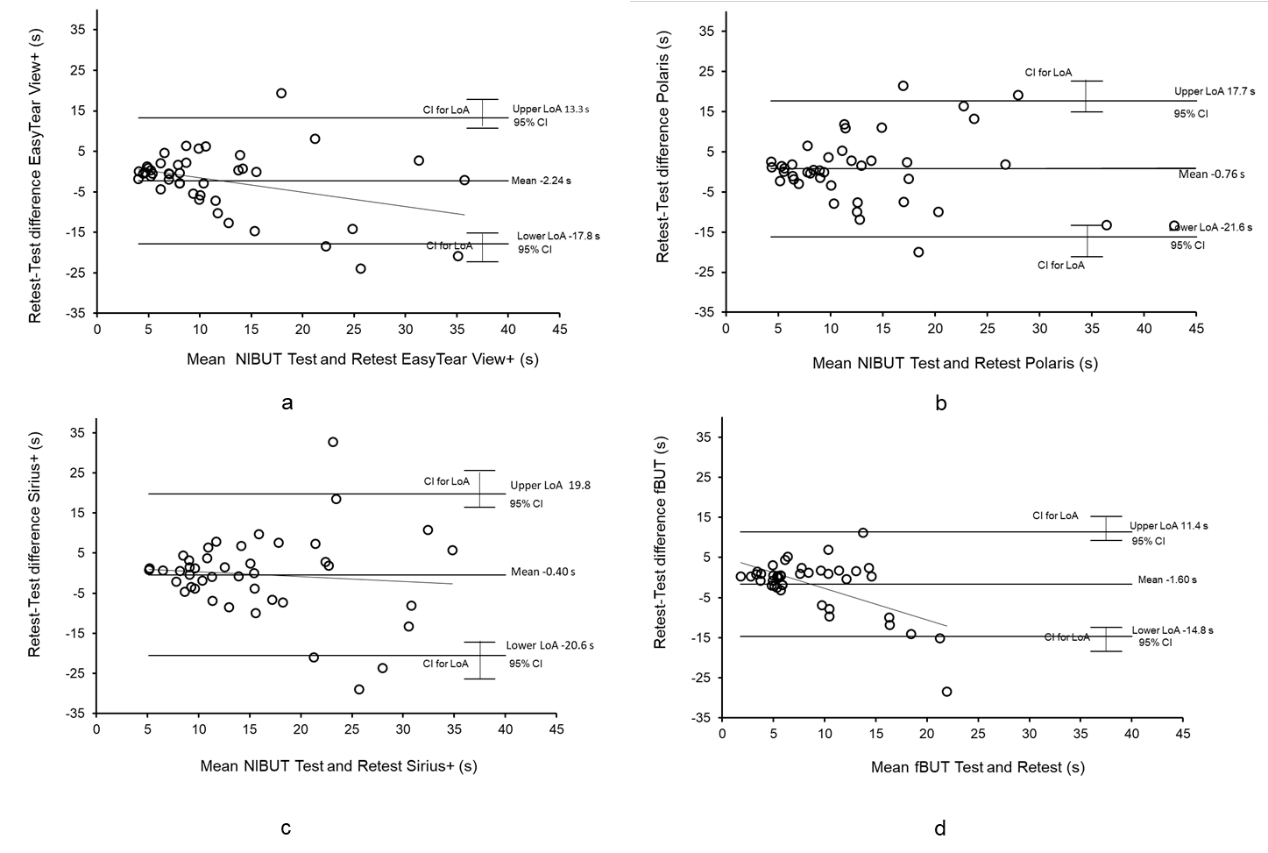


Figure S2: Bland–Altman plot for the test–retest for the four measurements in left eye in the first experiment of the study. The limits of agreement were calculated as mean ± 1.96 SD. Spearman Rho (correlation between the mean of test-retest and the difference between retest-test) resulted -0.22, -0.06, -0.002 and -0.16 (all n.s) for EasyTear View+ (a), Polaris (b), Sirius+ (c), and fBUT (d) respectively.


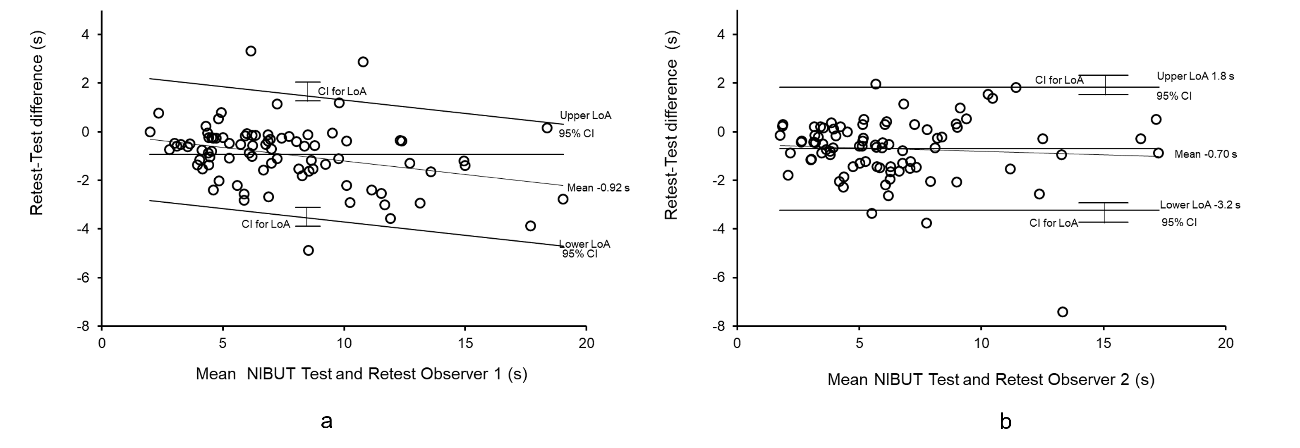


Figure S3: Bland–Altman plot for the test–retest for observer 1 and 2 in the second experiment of the study. The limits of agreement were calculated as mean ± 1.96 SD. Spearman Rho (correlation between the mean of test-retest and the difference between retest-test) resulted significant for the observer 1 (r=-0.29; p=0.008) whereas not significant for observer 2 (r=-0.04; p=0.74)
